# Evidence for G-quadruplex in the promoter of VEGFR-2 and its targeting to inhibit tumor angiogenesis

**DOI:** 10.1093/nar/gku1217

**Published:** 2014-12-16

**Authors:** E. Salvati, P. Zizza, A. Rizzo, S. Iachettini, C. Cingolani, C. D'Angelo, M. Porru, A. Randazzo, B. Pagano, E. Novellino, M.E. Pisanu, A. Stoppacciaro, F. Spinella, A. Bagnato, E. Gilson, C. Leonetti, A. Biroccio

Nucleic Acids Res. 2014 Mar;42(5):2945–57. doi: 10.1093/nar/gkt1289.

In Figure 3 of the above article, the authors have inadvertently taken the microscopic field of the VEGFR-2/VEGF-A from the empty/VEGF-A sample. A new Figure 3, showing representative images from a new experiment, is presented below. This error does not affect the findings or conclusion of the article.

The authors wish to apologise to the readers and publisher for this error.

**Figure 3.**
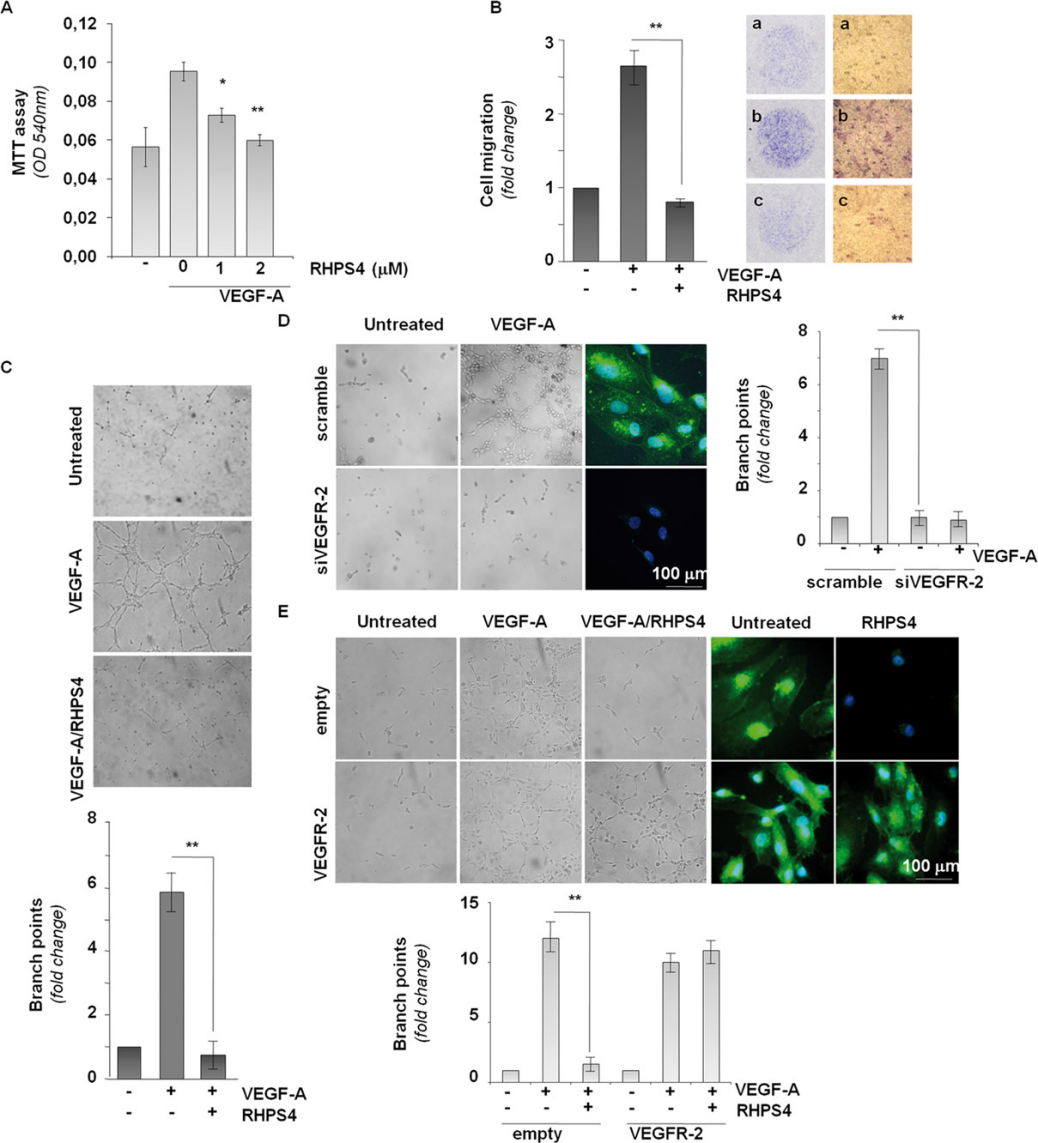
G4 stabilization impairs *in vitro* angiogenesis. **(A)** HUVEC cells, untreated (-) or treated with the indicated concentrations of RHPS4 for 72 hrs to achieve VEGFR-2 down-regulation, were starved for 24 hrs and then stimulated with VEGF-A in presence or absence of RHPS4. Cell viability was evaluated by MTT assay. Histograms represent the absorbance values at OD 540nm. **(B)** HUVEC cells, untreated (-) or treated with 0.5 μM RHPS4 for 72 hrs, were starved for 24 hrs in presence or absence of RHPS4 and then processed for chemotaxis assay. Histograms represent the fold change of the number of migrated cells in VEGF-A stimulated *vs* unstimulated condition. Pictures show migrated cells through the boyden chamber filter (magnification: 1X, left panels, and 40X, right panels) in untreated (a), VEGF-A stimulated (b) and VEGF-A/RHPS4 treated (c) cells. **(C)** HUVEC cells treated as in B were processed for the *in vitro* angiogenesis assay. The upper panel shows representative images of tubular structures (TS) in the indicated samples (20X magnification). Quantification of TS is reported in the lower panel. Histograms represent the mean number of branch points per field. **(D and E)** HUVEC cells transfected as indicated and treated as in B were processed for the *in vitro* angiogenesis assay. Representative images of TS are shown. IF pictures show VEGFR-2 staining merged with Hoechst in the indicated conditions (63X magnification). Histograms represent quantification of TS. Histograms show the mean values of three independent experiments while images show one representative of three independent experiments with similar results. Bar scale = Bars indicate means ±SD. * = *P* < 0.1 ** = *P* < 0.01.

